# Genomics-guided discovery and structure identification of cyclic lipopeptides from the *Bacillus siamensis* JFL15

**DOI:** 10.1371/journal.pone.0202893

**Published:** 2018-08-31

**Authors:** Ben-Hong Xu, Ya-Qin Lu, Zhi-Wei Ye, Qian-Wang Zheng, Tao Wei, Jun-Fang Lin, Li-Qiong Guo

**Affiliations:** 1 College of Food Science and Institute of Food Biotechnology, South China Agricultural University, Guangzhou, China; 2 Research Center for Micro-Ecological Agent Engineering and Technology of Guangdong Province, Guangzhou, China; Mizoram University, INDIA

## Abstract

In this research, a strain with broad-spectrum antimicrobial activities was isolated from the gastrointestinal tract of hairtail (*Trichiurus haumela*) and identified as *Bacillus siamensis* JFL15 through morphological, 16S rRNA, and average nucleotide identity analyses. The genome of *B*. *siamensis* JFL15 was sequenced, and three gene clusters involved in the biosynthesis of surfactin (*srf*), bacillibactin (*dhb*), and fengycin (*fen*) were predicted through antiSMASH analysis. The combined genomics-metabolics profiling of the strain revealed 20 active compounds, which belong to four main types of cyclic lipopeptides produced by *Bacillus* species: bacillibactin, iturin, fengycin, and surfactin. Among these lipopeptides, two high-purity antifungal components, namely, components b and c, were successfully identified as iturin A and bacillomycin F. The minimum inhibitory concentrations (MICs) of iturin A for *Magnapothe grisea*, *Rhizoctorzia solani*, and *Colletotrichum gloeosporioides* were 125.00, 62.50, and 125.00 μg/ml, respectively, whereas the MICs of bacillomycin F for these three organisms were 62.50, 31.25, and 62.50 μg/ml, respectively. The mechanism of bacillomycin F and iturin A against *M*. *grisea* was also investigated. Scanning electron microscopy (SEM) indicated that the surface of the hypha treated with iturin A or bacillomycin F became sunk, lumpy, and wrinkled. The diversity of the identified and predicted compounds from *B*. *siamensis* JFL15 suggested that this strain might be a promising biocontrol agent for an effective and environmentally friendly control of pathogenic microorganisms. To the best of our knowledge, this study is the first to describe cyclic lipopeptides purified and identified from *B*. *siamensis*.

## Introduction

*Bacillus* produces a distinguished class of cyclic lipopeptides (CLPs), which are known for their broad-spectrum antimicrobial activity, low toxicity, and high effectiveness under extreme conditions[1]. In contrast to conventional antibiotics, CLPs cause lethal effects on pathogenic bacteria by disrupting biological membranes through permeabilization; as a result, pathogenic bacteria experience greater difficulty in developing resistance to CLPs than to traditional antibiotics[2,3].

CLPs, which are considered as strong alternatives for conventional antibiotics, are divided into three main families, and they have a common amphipathic structure with a hydrophilic peptide portion and a hydrophobic fatty acid portion[4,5]: (1) surfactin family members are cyclic heptapeptides linked to a β-hydroxy fatty acid chain between C13 and C17; (2) fengycin family members, including plipastatin and fengycin, are cyclic octapeptide-containing decapeptides linked to a β-hydroxy fatty acid chain between C12 and C19; and iturin family members, such as iturin, mycosubtilin, and bacillomycin, are cyclic heptapeptides linked to a β-amino fatty acid chain between C15 and C18[6,7]. Fengycin and iturin exhibit high antifungal activities against numerous phytopathogens. Surfactin is mostly known for its strong antibacterial and antiviral activities[8].

CLPs are synthesized by a large multifunctional non-ribosomal enzyme complex called nonribosomal peptide synthetase (NRPS), which confers considerable structural diversity to molecules and results in the production of linear, branched, or cyclic compounds[9]. In a typical NRPS module, at least three essential domains are present: (i) a catalytic domain that selects a specific monomer, (ii) a carrier protein domain that assists the attachment of a monomer after thioesterification, and (iii) a second catalytic domain that participates in chain elongation[10]. *srfA* operon for the synthesis of surfactin, which is the most well-studied lipopeptide at a genetic level, encodes three NRPSs (*srfAA*, *srfAB*, and *srfAC*) and a thioesterase/acyltransferase enzyme (*srfAD*)[9,11].

The development of new CLPs involves the cultivation of microorganisms, chemical extraction of metabolites, and elucidation of final structures. This bioactivity-guided approach previously facilitated the discovery of many valuable chemicals; however, their rediscovery often reveals that they are known metabolites, and new molecules are hardly identified[12]. Demands for novel classes of potent antibiotics with different mechanisms have also remained unaddressed because of the prevalence of antibiotic-resistant pathogens. Nevertheless, the improvement of cost-effective and high-throughput sequencing technology and the remarkable increase in the number of bacterial genomes have considerably contributed to natural product research[13]. This strategy is commonly referred to as genome mining, which is divided into two steps; in the first step, “talented” microbes that may produce biomolecules with novel bioactivities are identified according to the genome sequence analysis and subsequent characterization of biosynthetic gene clusters (BGCs)[10]. This approach also helps identify genomic entities likely responsible for the production of new molecules.

In the present work, a strain that exhibited a strong antimicrobial activity against pathogenic microorganisms was isolated and identified. Whole-genome sequencing revealed that this strain is *Bacillus siamensis* JFL15. Moreover, several CLP products of this strain were determined through the analysis of secondary metabolite BGCs by using antiSMASH software. These products were subsequently isolated, purified, and characterized through chromatographic, LC-MS/MS, and other chemical analytical techniques. The antimicrobial effects and mechanisms of these purified compounds were further evaluated.

## Materials and methods

### Microorganisms and culture conditions

Bacterial strain was isolated from the gastrointestinal tract of hairtail (*Trichiurus haumela*)[14]. The indicator fungi *Magnaporthe grisea* and *Rhizoctonia solani* were kindly provided by Professor Erxun Zhou at South China Agricultural University, and the indicator fungi *Colletotrichum gloeosporioides* and bacteria *Vibrio harveyi* were stored in our labotatory. Luria-Bertani (LB) broth medium (containing 10 g/L tryptone, 5 g/L yeast extract, and 5 g/L NaCl in distilled water) was used as the growth medium for the strain JFL15 and *V*. *harveyi*, and mineral salt medium (MSM) (containing 20 g/L sucrose, 2 g/L NH_4_NO_3_, 3 g/L KH_2_PO_4_, 10 g/L Na_2_HPO_4_, 0.2 g/L MgSO_4_, 0.2 g/L yeast extract, 0.7 μg/L CaCl_2_, and 1 μg/L MnSO_4_ in distilled water) was used for production of antifungal compounds by culturing at 30°C for 3 days with continuous shaking at 200 rpm. The indicator of pathogenic fungi were incubated on PDA plate at 28°C for 7 days.

### DNA isolation, genome sequencing and assembly

Genomic DNA of the strain JFL15 was isolated and sequenced using a whole-genome shotgun strategy. All data were generated by paired-end sequencing of cloned inserts with two different insert sizes (500 bp, 6000 bp) using Illumina Hiseq2000 Sequencer at BGI-Shenzhen. After removing the low complexity, low quality, adapter and duplication contamination raw reads, the clean reads were assembled into contigs and scaffolds using the whole-genome *do novo* assembler SOAPdenovo2.04 with optimal assembly acquired with the key parameter K = 103.

### Genome annotation

Coding sequence (CDS) prediction was carried out with the Glimmer version 3.0 and MAKER pipeline prediction system[15]. The functionally annotation was accomplished by BlastP analysis of sequences in the NCBI nr, SwissProt and KEGG databases (parameters: E-value: < 1E-5, identity > 40%, coverage > 60%) and by manual curation of the outputs of a variety of similarity searches[16]. Each gene was functionally classified into functional terms, including GO, COGs, and KEGG pathways. Non-coding RNAs were predicted by rRNAmmer 1.2[17], tRNAscan-SE 1.2[18], and Rfam 10.1[19]. The G+C content was calculated using the genome sequence. The genome sequence of JFL15 was deposited in the GenBank database under the accession number of LFWQ00000000, and the BioProject and BioSample ID in GenBank is PRJNA288238 and SAMN03796075, respectively.

### Phylogenetic and genome comparative analysis

A phylogenetic tree based on the 16S rDNA sequences was constructed with the neighbour-joining method using the software MEGA5.0, the 16S rDNA sequences of other closely-related *Bacillus* species were obtained from the EzTaxon-server (http://eztaxon-e.ezbiocloud.net/). Bootstrap values on the bifurcating branches were performed using 1,000 replications for the phylogenetic tree.

The complete genome sequence of a microbial strain is the most fundamental information for microbial taxonomy. Average nucleotide identity (ANI), which represents the average identity values between two homologous genomes of prokaryotic strains, was proposed almost ten years ago and has become a possible next-generation gold standard for species delineation[20]. It is now generally accepted that ANI values of 95–96%, which is equivalent to a DNA–DNA hybridization cut-off value of 70%, can be taken as a boundary for species delineation[21]. In order to obtain accurate taxonomic status of strain JFL15, genomic comparison of JFL15 and other reference genomes was performed by the JSpecies software package which was used to calculate the Mummer-based ANI (ANIm) using the default conditions previously described. Reference genomes for comparison purposes were available in the GenBank database.

### Identification of BGCs of CLPs in the strain JFL15

The software tool antiSMASH (http://antismash.secondarymetabolites.org) was used to predict putative NRPS genes involved in CLPs synthesis and detail structures of CLPs. The results obtained from genomic sequences correlated with NRPS pathway consisted of detailed functional domain annotation, predicted core structure, and levels of genomic identity to known BGCs catalogued in the Minimum Information on Biosynthetic Gene Cluster (MIBiG).

### Antimicrobial assay

The antifungal activity of the extracted metabolites were determined by Oxford Cup Method[22]: Hyphae discs of phytopathogens (*M*. *grisea*, *R*. *solani* and *C*. *gloeosporioides*) were placed in the center of each PDA plate, then the sterilized oxford cup was put on the plate, which was 3 cm away from the edge of the mycelial colony. 150 μL of each CLP was added into the oxford cup, then incubated for 7 days at 28°C. The same volume of methanol was used as control. The antifungal effect was determined by the semidiameter of inhibition zone. In the analysis of antibacterial activity, 150 μL of each extract was added into oxford cup. Test cultures of *V*. *harveyi* was plated from the liquid cultures on solid LB medium, dried for 20 minutes prior applying the extracts. Plates were incubated overnight at 37°C.

The zones of inhibition were measured manually with accuracy ± 1 mm. All experiments were conducted in triplicate.

### Production and purification of CLPs

After shaking incubation in 5 L of MSM medium at 30°C for 3 days, the cell-free supernatant was obtained after 8,000 ×*g* centrifugation for 10 min at 4°C, and the pH was adjusted to 2.0 with 6M HCl and stored overnight at 4°C. The precipitate was collected by centrifugation at 8,000 ×*g* for 10 min at 4°C, washed 3 times with acidic water (pH 2.0), and neutralized with 6M NaOH before freeze-dried in vacuum. The powder was extracted 5 times with methanol for 3 hr. The brown-colored extract was concentrated using a rotary evaporator under reduced pressure.

Extracts were fractionated in two steps: size-exclusion chromatography and preparative HPLC. The concentrate was subjected to a Sephadex LH-20 gel filtration column, which was equilibrated and eluted with methanol at a flow rate of 0.8 mL/min. The eluent was collected (4 mL/tube), and the absorbance at 214 nm was measured using a UV spectrophotometer (methanol as CK). The active fractions were pooled and concentrated using a rotary evaporator.

Further purification was carried out by preparation HPLC (SHIMADZU LC-8A, Japan) with a C18 column (250 mm × 4.6 mm, 5 μm, Phenomenex, USA) at room temperature. The mobile phase consisted of solvent A (acetonitrile) and solvent B (0.1% trifluoroacetic acid (TFA) in water). A linear gradient was used for elution at a flow rate of 10 mL/min as follows: 0–30 min, from 10% to 50% B (linear gradient); 30–50 min, from 50% to 93% B (linear gradient); 50–70 min, 93% B (isocratic). Elution was monitored by determining absorbance at 214 nm. Fractions with the highest antimicrobial activity were selected for structural identification.

### LC-MS/MS analysis

All compounds with strong antimicrobial activity were obtained in pure and then analyzed in positive ion mode using a quadrupole time-of-flight mass spectrometry (Q-TOF-MS) system (Agilent Technologies 6540B, USA). This system was equipped with an ultra-high performance liquid chromatography (UHPLC), an ESI interface, a collision cell, and two mass analyzers. The operating parameters included a capillary voltage of 800 V, cone voltage of 40 V, fragmentor voltage of 175 V, and capillary temperature of 27°C.

### MICs and SEM analysis

Two high purity antifungal substances (bacillomycin F and iturin A) from 2.6 were selected for MIC and SEM analysis. The MIC of two antifungal substances against three phytopathogens was determined by agar-well diffusion method as described 、[22]. A range of concentrations (15.63, 31.25, 62.50, 125.00 and 250.00 μg/mL) of bacillomycin F and iturin A were added into the oxford cups. After incubation for 7 days at 28°C, the mycelial growth inhibition was determined by measuring the semidiameter of the inhibition zone. The same volume of methanol was used as a control, and the experiments were conducted in triplicate.

100 μL of hyphae suspension of *M*. *grisea* were added into PDA liquid medium, which contained a range of concentrations (15.63, 31.25, 62.5, 125 and 250 μg/mL) of two different antifungal substances, respectively. PDA liquid medium with no antifungal substances was regarded as control. After shaking incubation at 28°C for 3 days, *M*. *grisea* mycelia was harvested by centrifugation at 4°C. The mycelia for SEM were washed by isotonic sterile saline water (0.85% NaCl) and then fixed with glutaraldehyde[23]. The experiments were conducted in triplicate.

## Results

### Morphological characteristics and general genome features of *Bacillus* sp. JFL15

The strain JFL15, which is a strict aerobic, Gram-positive, motile, and endospore-forming strain growing at 45°C and tolerating up to 7.5% NaCl, was isolated from the gastrointestinal tract of hairtail (*Trichiurus haumela*). The original information of this strain and its genome sequencing project were determined according to the minimum information about a genome sequence (MIGS) recommendations shown in S1 Table.

The complete genome sequence of strain JFL15 is characterized by a circular chromosome of 3,841,225 bp with a 46.36% G+C content. No plasmids were identified, but 3600 protein-coding DNA sequences, 82 tRNA genes, 25 rRNA operons, and 9 sRNA genes were predicted. In addition, 123 tandem repeat sequences were determined by the Tandem Repeat Finder. The features of the assembled genome sequences are shown in Table 1.

**Table 1:** Comparison of genomic features of the *Bacillus* JFL15 with genomes of other *Bacillus* spp.

Features	*Bacillus* JFL15	*B*. *siamensis* KCTC 13613 ^T^	*B*. *velezensis* FZB42^T^	*B*. *amyloliquefaciens* DSM7^T^	*B*. *subtilis* 168 ^T^
**Genome size (bp)**	3,841,225	3,784,323	3,918,591	3,908,199	4,214,630
**G+C content (%)**	46.36	46.30	46.49	46.10	43.51
**Protein-coding sequences**	3600	3892	3693	3893	4106
**Percent of coding region**	88.52	n.a*	88.0	n.a	87.2
**rRNA operons**	25	n.a	10	10	10
**tRNA genes**	82	n.a	89	94	86

*n.a: not applicable.

### Phylogenetic and genome comparative analysis

**Fig 1:**
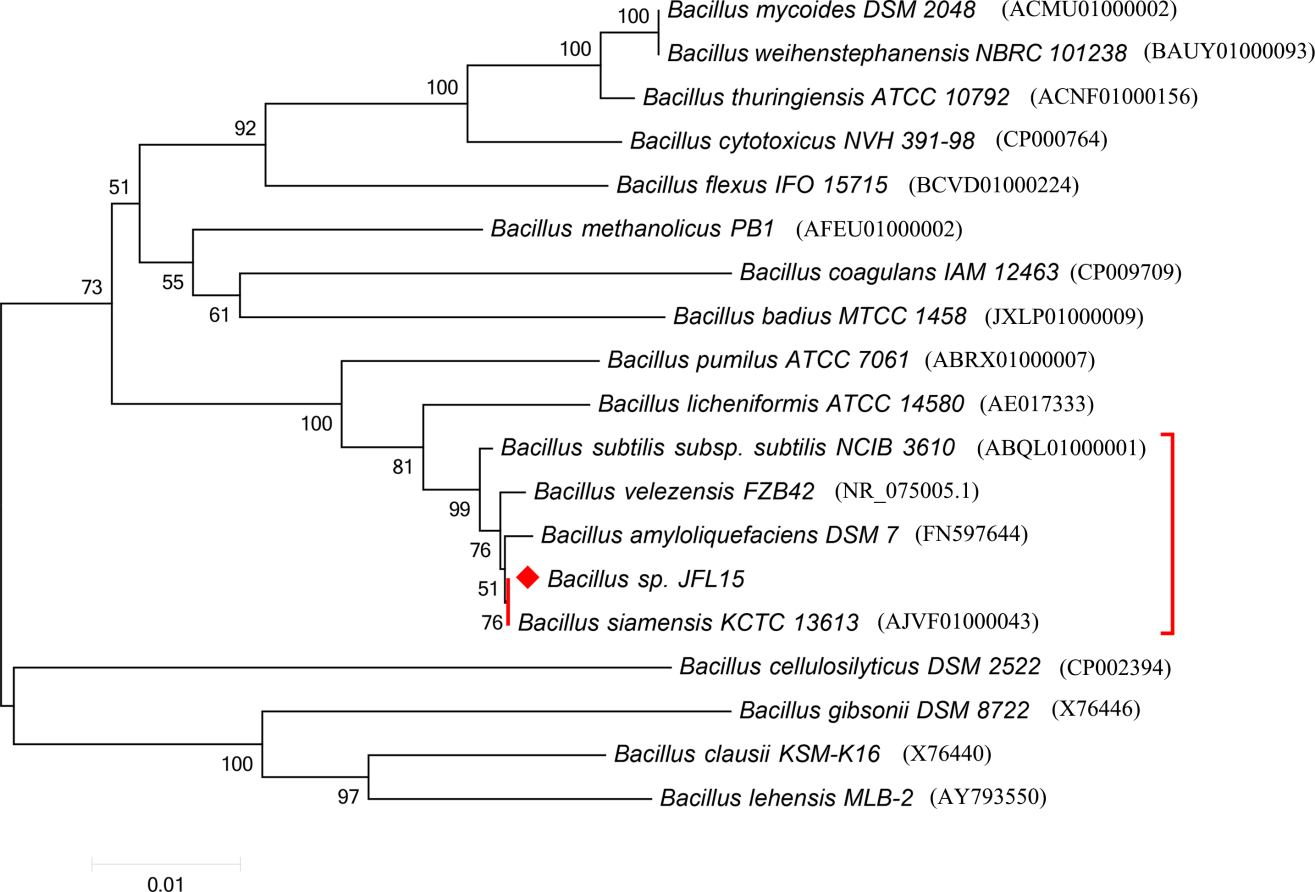
Phylogenetic tree based on 16S rRNA sequences of strain JFL15 and other *Bacillus* species. The tree was constructed by the neighbor-joining method, and the Bootstrap values of ≥ 50% are shown at the branching points. Bar, 0.01 substitutions per nucleotide position.

The almost complete 16S rRNA gene sequence (1538 bp) of strain JFL15 was determined, and the 16S rRNA sequences of the type strains of species closely related to the JFL15 were downloaded from the EzTaxon database. A phylogenetic tree based on 16S rDNA was reconstructed by the neighbor-joining method in MEGA5.0. Phylogenetic analysis (Fig 1) indicated that the isolated strain was identified as a member of the genus *Bacillus*, which consists of the type strains of *B*. *siamensis* (98.05% 16S rRNA gene sequence similarity), *B*. *velezensis* (97.85%), *B*. *amyloliquefaciens* (97.79%), and *B*. *subtilis* (97.66%). Many of these *Bacillus* species exhibit extremely high 16S rRNA gene sequence similarity. However, the accurate taxonomic status of these strains is difficult to be obtained by 16S rRNA. Therefore, to characterize the taxonomic status of these strains accurately, we performed an ANI analysis based on their complete genome sequence with enhanced precision. The results showed that strain JFL15 and *B*. *siamensis* KCTC 13613^T^ displayed an ANI of 98.73% (Table 2). *B*. *amyloliquefaciens* DSM7^T^, *B*. *velezensis* FZB42^T^, and *B*. *subtilis* 168^T^ respectively displayed ANI values of 93.82%, 94.23%, and 76.45% compared with that of strain JFL15. The recommended ANI values for species delineation were 95%–96%, which corresponded to a DNA–DNA hybridization cut-off point of 70%. Thus, the strain was identified as *B*. *siamensis* JFL15.

**Table 2:** ANI analysis of type strains from the *Bacillus* group.

Strain	ANI values against the target genomes				
	JFL15	DSM7^T^	FZB42^T^	168 ^T^	KCTC 13613 ^T^
***Bacillus* JFL15**	-	93.53	94.25	76.23	**98.73**
***B*.*amyloliquefaciens* DSM7**^**T**^	93.82	-	94.04	76.44	93.83
***B*. *velezensis* FZB42**^**T**^	94.23	93.71	-	76.45	94.24
***B*. *subtilis* 168** ^**T**^	76.45	76.33	76.52	-	76.43
***B*.*siamensis* KCTC 13613** ^**T**^	**98.71**	93.53	94.2	76.16	-

Figures in bold denote strains belonging to the same genomospecies.

### Functional gene annotation

#### Function and classification of COG

All of the predicted protein sequences of *B*. *siamensis* JFL15 were compared with those in the COG database to search for homologous amino acid sequences in the database.

**Fig 2:**
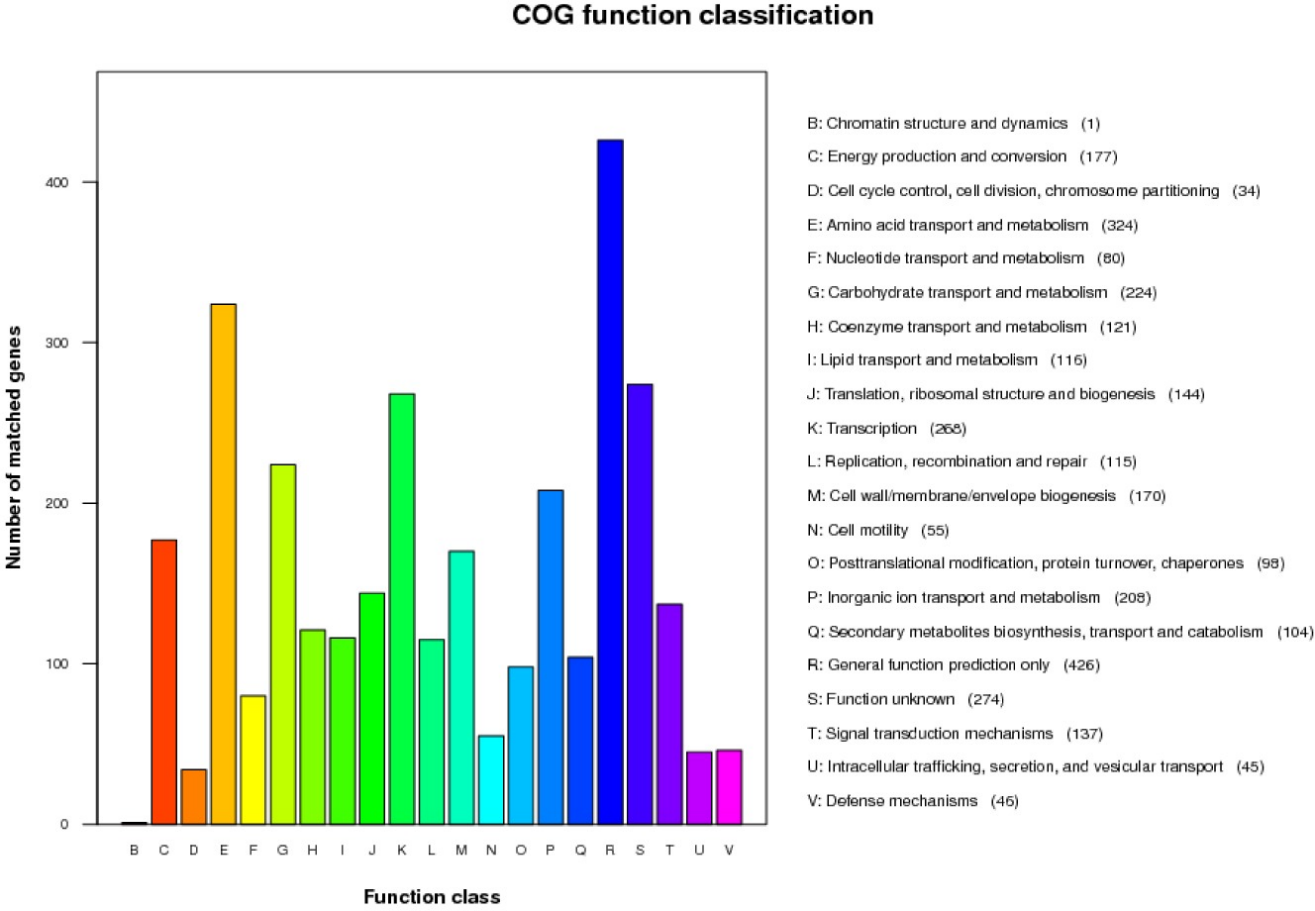
The COG function annotation of *B*. *siamensis* JFL15. Distribution of Genes in different COG function classification.

Each protein was assigned with a COG number when it was functionally annotated, and each COG number represented a class of protein. The proteins were then subjected to functional clustering analysis according to the classification criteria of COG function (Fig 2). In *B*. *siamensis* JFL15, 3167 protein sequences had COG numbers, accounting for 87.97% of all protein sequences. The percentage of functional proteins in the total number of chromosomes was the highest, which was 13.45%. Most of the proteins were involved in amino acid transport and metabolism (R), carbohydrate transport and metabolism (G), and transcription (K), and these proteins accounted for 10.23%, 7.07%, and 8.46% of the total protein sequence, respectively. The metabolic activity of the amino acids and sugars of *B*. *siamensis* JFL15 was high. A total of 104 proteins involved in the secondary metabolite biosynthesis, transport, and catabolism (Q) of *B*. *siamensis* JFL15 constituted 3.28% of the total protein sequence. Therefore, *B*. *siamensis* JFL15 synthesized high amounts of secondary metabolites, especially antimicrobial substances.

#### Function and classification of GO

**Fig 3:**
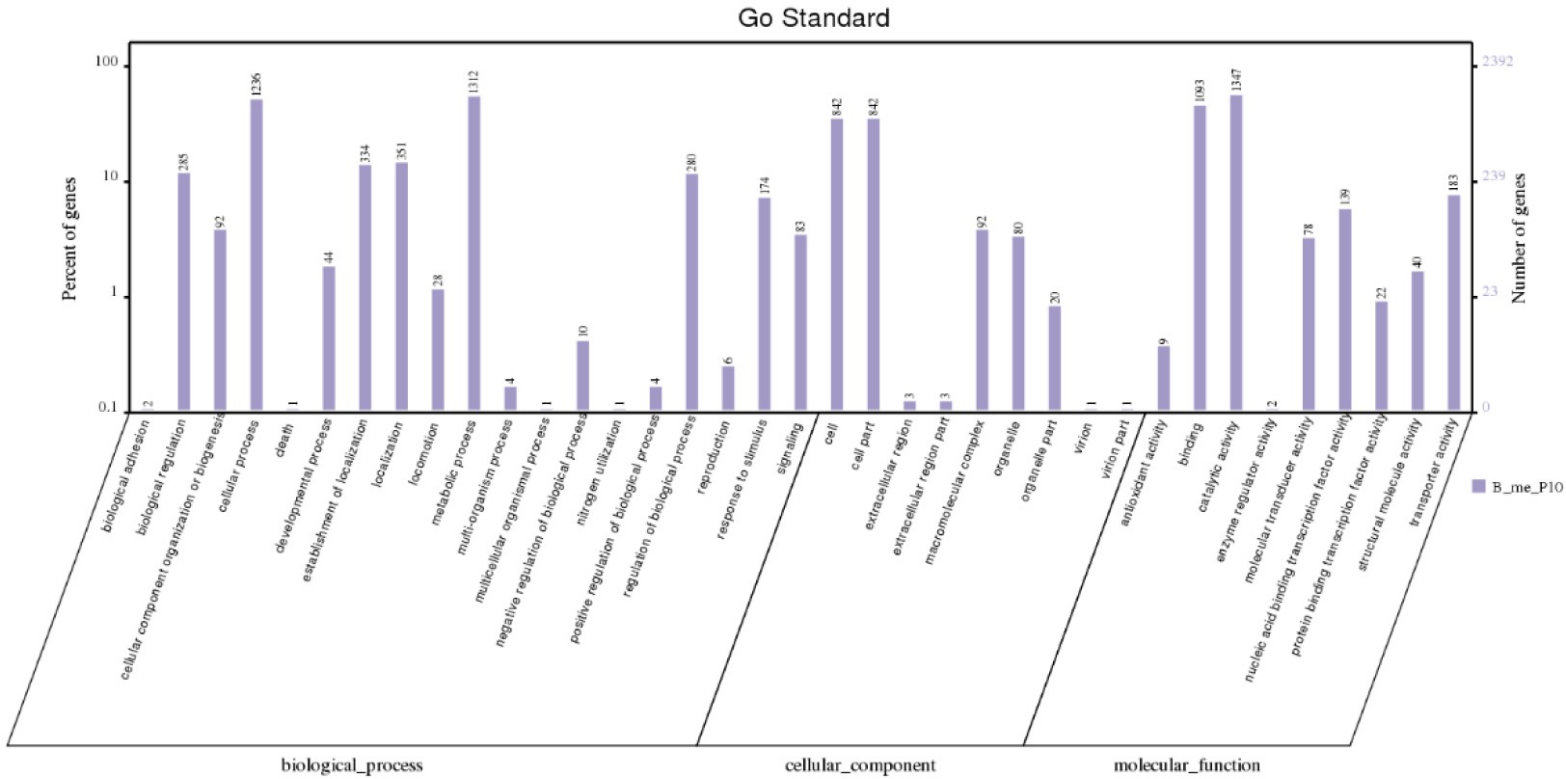
The GO function annotation of *B*. *siamensis* JFL15. Distribution of Genes in different GO function classification.

The Interproscan database is a non-redundant database developed by European Bioinformatics Institute (EBI) that integrates protein families, domains, and functional sites. In this study, this database was used to predict all of the protein domains and functional sites of *B*. *siamensis* JFL15 and extract the information of GO. The WEGO online tool was then utilized to perform GO function classification (Fig 3). GO function classification includes three aspects: biological process, cellular component, and molecular function. Each aspect comprises a number of small branches. Cellular and metabolic processes were the most active in the biological process. Cell and cell parts have a considerable advantage in cells. Binding and catalytic activity play a prominent role in molecular function.

#### Analysis of the metabolic pathway of KEGG

**Fig 4:**
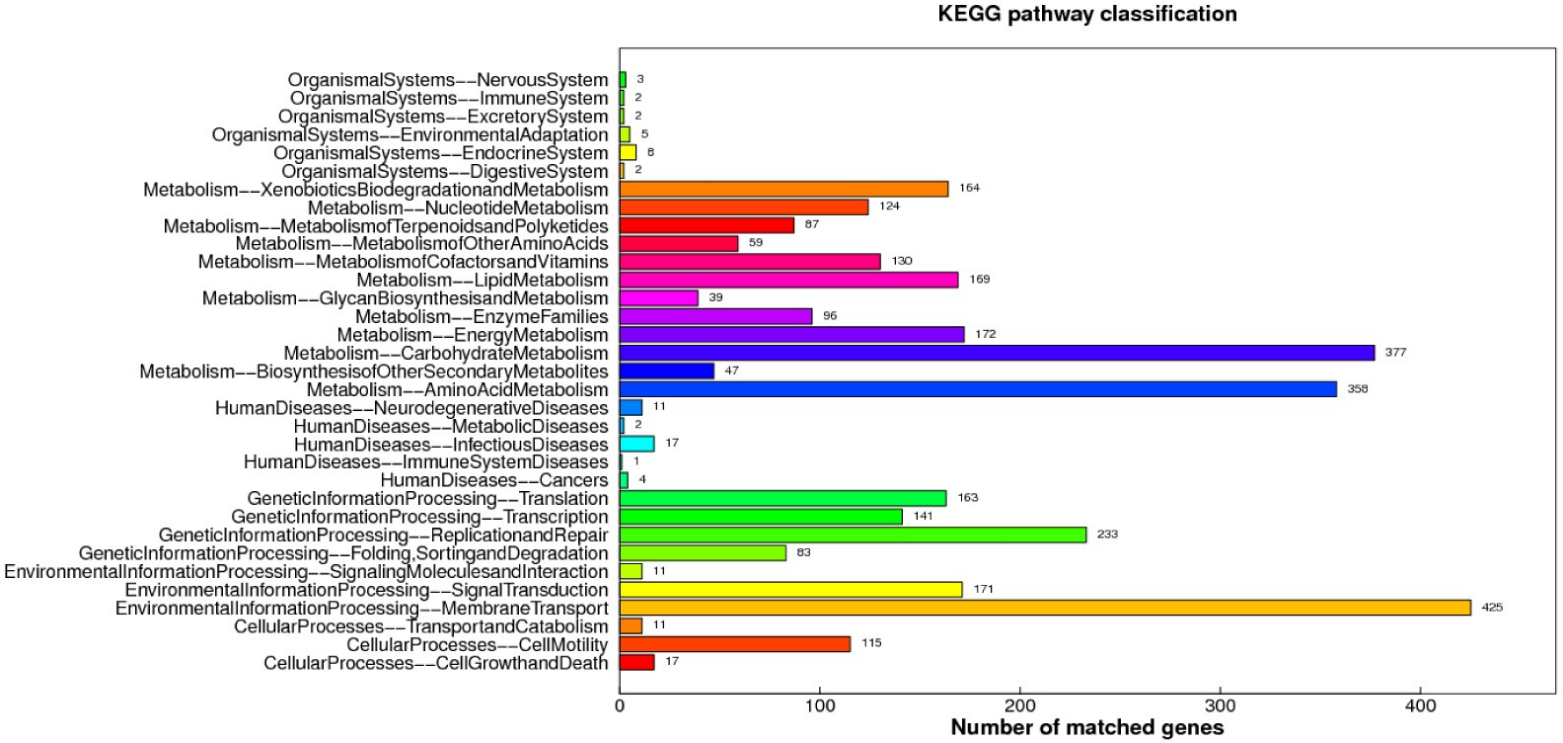
The KEGG function annotation of *B*. *siamensis* JFL15. Distribution of Genes in different KEGG categories.

The genes involved in the metabolic pathways were analyzed statistically by using the KEGG analysis tool in the genome sequence of *B*. *siamensis* JFL15. In Fig 4, the genome of *B*. *siamensis* JFL15 mainly included genes associated with xenobiotic biodegradation and metabolism, nucleotide metabolism, terpenoid and polyketide metabolism, amino acid metabolism, cofactor and vitamin metabolism, lipid metabolism, glycan biosynthesis and metabolism, enzyme families, energy metabolism, carbohydrate metabolism, and secondary metabolite biosynthesis. A total of 425 genes participated in membrane transport, followed by glucose metabolism (377) and amino acid metabolism (358). Furthermore, 47 genes were involved in secondary metabolite metabolism. The abundant metabolic pathway provided the necessary carbon and nitrogen sources for the growth and secondary metabolite synthesis of *B*. *siamensis* JFL15. In addition, a large number of genes implicated in membrane transport might be associated with extracellular metabolism.

### Analysis of BGCs of CLPs in *B*. *siamensis* JFL15

**Fig 5:**
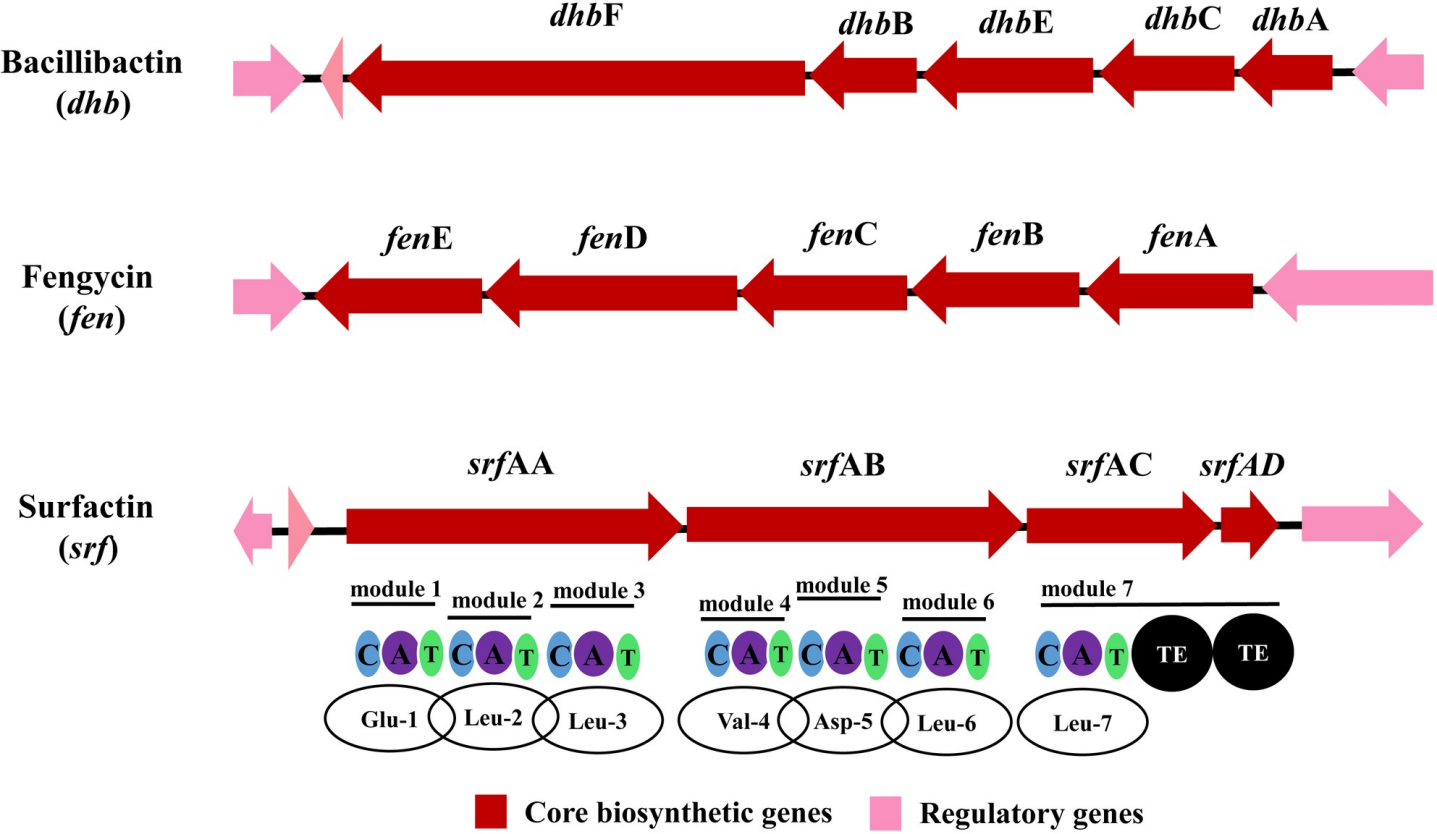
Three CLPs gene clusters identified in *B*.*siamensis* JFL15. Arrows indicate gene clusters. Genes highlighted in red represent the core biosynthetic genes and pick represent the regulatory genes. The domains are labelled by: C, condensation; A, adenylation; T, thiolation and TE, thioesterification.

According to the antiSMASH4.0 analysis of *B*. *siamensis* JFL15 genome, 30 gene clusters were predicted to be involved in the secondary metabolism of the strain, and 3 gene clusters were responsible for the biosynthesis of bioactive CLPs via NRPSs: surfactin (*srf*), bacillibactin (*dhb*), and fengycin (*fen*), with known antagonistic activities (Fig 5). The sequences associated with the production of nonribosomal peptides, namely, surfactin, bacillibactin, and fengycin, corresponded to 78%, 100%, and 100% of the identified gene clusters, respectively (Table 3), suggesting that *B*. *siamensis* JFL15 could produce a new kind of surfactin. The gene cluster of surfactin biosynthesis was further analyzed, and the results revealed that this cluster consisted of a 7th module containing the classical C-A-T tridomain architecture, a termination Te domain, and an ACPS domain. The amino acid backbone structure of the potential peptide was predicted as Glu-Leu-Leu-Val-Asp-Leu-Leu based on the binding specificities of the A domains associated with the aforementioned NRPS clusters (Fig 5).

**Table 3:** The predicted gene clusters related to synthesis of antibiotics in *B*. *siamensis* JFL15.

Gene cluster type	Antibiotics	Size (Kb)	Identity (%)
**NRPS**	Bacillibactin	66.779	100
	Surfactin	65.419	78
	Fengycin	137.830	100
**PKS**	Difficidin	100.553	53
	Bacillaene	102.610	100
**Others**	Plantazolicin	34.198	91
	Butirosin	41.244	7

Three gene clusters with polyketide synthase (PKS) genes within the genome of *B*. *siamensis* JFL15 are involved in bacillaene (*bae*) and difficidin (*dfn*) biosynthesis. Two other clusters for the antibiotics plantathiazolicin and butirosin synthesized in other *Bacillus* strains were found in the *B*. *siamensis* JFL15 genome (Table 3). Furthermore, a putative NRPS cluster of eight genes probably encoding a novel antibiotic was found in the chromosome of *B*. *siamensis* JFL15. As a result, *B*. *siamensis* JFL15 shows great capability for antibiosis. A previous investigation involving *in vitro* assays also showed that this strain can antagonize several pathogens.

### Purification and LC-ESI analysis of CLPs from *B*. *siamensis* JFL15

**Fig 6:**
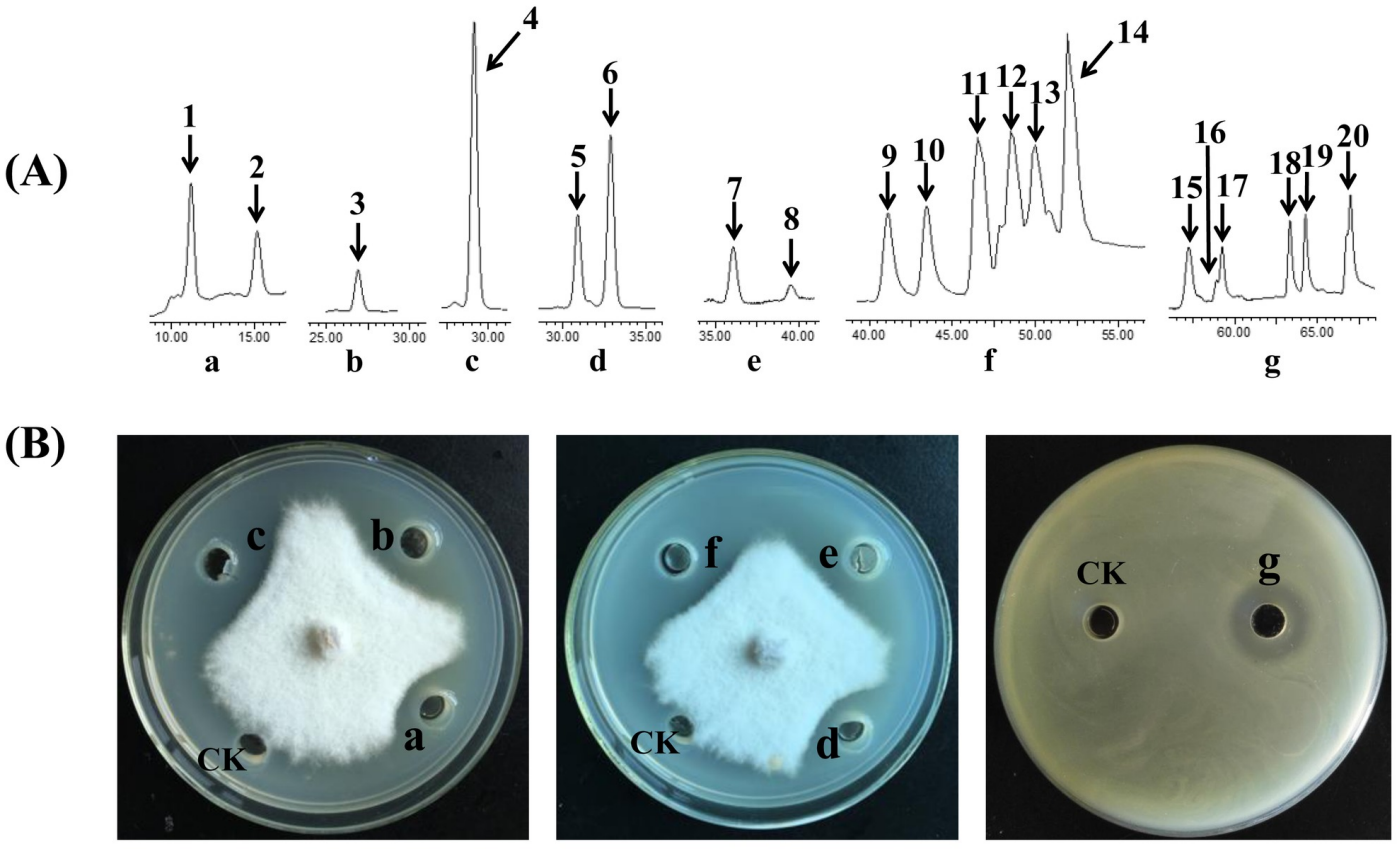
HPLC and antimicrobial activity analysis of the purified substances from *B*. *siamensis* JFL15. a, b, c, d, e, f, and g represent different components purified from *B*. *siamensis* JFL15. The peaks with numerical labels were infused into the Q-TOF MS and fractionated for further analysis.

Antimicrobial compounds were isolated through HCl precipitation from 5 L of the cell-free supernatants of *B*. *siamensis* JFL15 culture, Sephadex LH-20 chromatography, and subsequent preparation reversed phase chromatography[24]. Six components (a, b, c, d, e, and f) contained 20 compounds with strong antimicrobial activities were purified (Fig 6A). The purities of components b and c, which contained one component, were higher purity than those of the other components. In addition to the high antibacterial activity of component f against *V*. *harveyi*, a strong antifungal activity against *C*. *gloeosporioides* was observed in the five other components (Fig 6B).

LC-ESI mass spectrometry was chosen to elucidate the accurate molecular weight of the purified compounds, and our results were compared with the mass data obtained in previous studies. We found that these 20 compounds belonged to four main types of cyclic lipopeptides produced by *Bacillus* species: bacillibactin, iturin, fengycin, and surfactin. The protonated molecular ion [M+H]^+^ and [M-H]^−^ peaks of all of the 20 compounds were detected (Table 4). All of these molecules within each class had a 14 Da difference in molecular weights, suggesting the presence of varied lengths of fatty acid chains within each group (CH_2_ = 14 Da). Several isomers also exist in iturin and surfactin families.

**Table 4:** The m/z value of CLPs detected by ESI-CID-MS.

Compound no.	Peak no.	m/z[M+H]^+^	m/z[M-H]^-^	M	Identified
**a**	1	883.2621	ND	882.2	Bacillibactin
	2	897.2750	ND	896.2	Bacillibactin
**b**	3	1043.5559	1041.5378	1042.5	C_14_Iturin A
**c**	4	1057.5669	1055.5541	1056.5	C_14_Bacillomycin F
**d**	5	1057.5714	1055.5516	1056.5	C_14_Bacillomycin F
	6	1071.5813	1069.5716	1070.5	C_15_Bacillomycin F
**e**	7	1071.5831	1069.5697	1070.5	C_15_Bacillomycin F
	8	1085.5959	1083.5845	1084.5	C_16_Bacillomycin F
**f**	9	1435.7693	1433.7581	1434.7	C_14_Fengycin A
	10	1449.7833	1447.7726	1448.7	C_15_Fengycin A
	11	1463.8011	1461.7898	1462.8	C_16_Fengycin A
	12	1477.8140	1475.8022	1476.8	C_15_Fengycin B
	13	1491.8315	1489.7865	1490.8	C_16_Fengycin B
	14	1505.8431	1503.8146	1504.8	C_17_Fengycin B
**g**	15	994.6423	992.6437	993.6	C_12_Surfactin
	16	1008.6574	1006.6458	1007.6	C_13_Surfactin
	17	1022.6717	1020.6603	1021.6	C_14_Surfactin
	18	1022.6665	1020.6654	1021.6	C_15_Surfactin
	19	1036.6887	1034.6779	1035.6	C_16_Surfactin
	20	1050.7052	1048.6918	1049.6	C_17_Surfactin

ND: Not detected.

### LC–MS/MS analysis of CLPs

Each compound was used for ESI-CID-MS analysis. In this study, components b and c containing one component were selected as examples. ESI-CID-MS analysis was performed using [M+H]^+^ (*m*/*z* 1043.56) and [M+H]^+^ (*m*/*z* 1057.57) as precursor ions to further confirm whether the antifungal substances were iturin A and bacillomycin F, respectively. The details of the masses are summarized in Table 5.

**Table 5:** Summary of the masses identified on LC-MS/MS analysis.

Compound no.	Retention time (min)	Mass obtained by LC-MS/ MS analysis		Compound identified
		Positive ionization (M+H^+^)	Characteristic fragment ions	
**b**	26.66	1043.56	212.1, 392.2, 638.4, 801.4, 915.5, 932.5	Iturin A
**c**	29.81	1057.57	423.2, 635.3, 652.4, 684.3, 781.3, 815.5, 929.5, 946.5	Bacillomycin F

**Fig 7:**
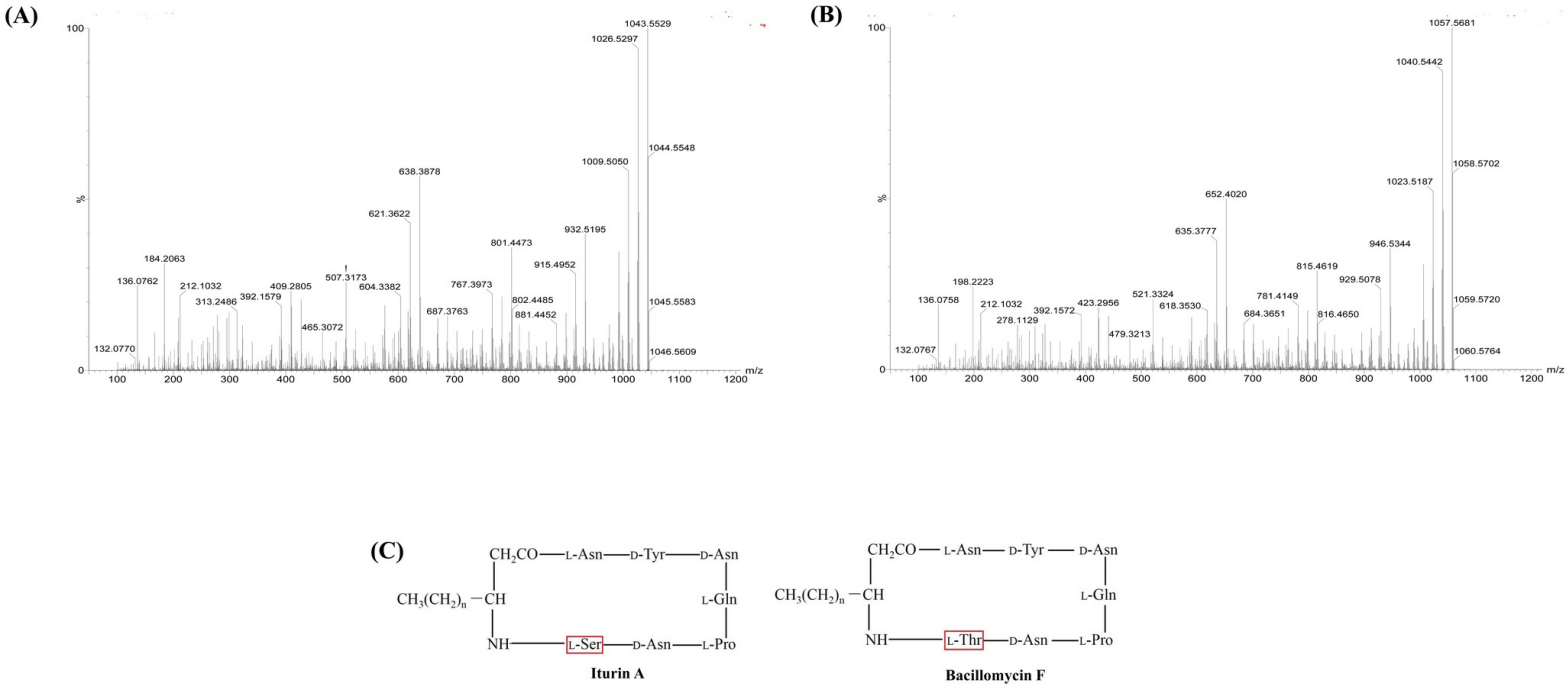
ESI-CID-MS analysis of iturin A (m/z 1043.56) and bacillomycin F (m/z 1057.57). The MS/MS spectrum of protons ions of iturin A at m/z 1043.56 (A) and bacillomycin F at m/z 1057.57 (B). The chemical structure of iturin A and bacillomycin F (C).

The fragmentation data of the specific masses based on LC–MS/MS were analyzed in detail to determine the presence of the fingerprint mass of each compound related to a structure reported previously. In Fig 7A, the detailed LC–MS/MS-based fragmentation analysis of the mass fragment ions with *m*/*z* 212.1, 392.2, 638.4, 801.4, 915.5, and 932.5, revealed that they were b- or y-type fragment ions generated by broken precursor ions [M+H]^+^ (*m*/*z* 1043.56). In comparison with literature data, results illustrate that the substance with a molecular weight at *m*/*z* 1043.56 was iturin A[25].

For component c (*m*/*z* 1057.5714, precursor ion), the typical (b-type or y-type) fragment ions at *m*/*z* 423.2, 635.3, 652.4, 684.3, 781.3, 815.5, 929.5, and 946.5 were detected in the CID spectrum (Fig 7B). These results are consistent with the mass fragments of bacillomycin F, which have been reported in a previous study [26].

### MIC and SEM analysis of iturin A and bacillomycin F against pathogenic fungi

The MICs of iturin A (component b) and bacillomycin F (component c) against *M*. *grisea*, *R*. *solani*, and *C*. *gloeosporioides* were determined through agar-well diffusion method. The two compounds exhibited different MICs against various phytopathogens (Table 6). The antifungal activity of iturin A was slightly higher than that of bacillomycin F.

**Table 6:** Antifungal activity of bacillomycin F and iturin A against phytopathogens.

Sample	MIC (μg/ml)		
	*M*. *grisea*	*R*. *solani*	*C*. *gloeosporioides*
**Iturin A**	62.50	31.25	62.50
**Bacillomycin F**	125.00	62.50	125.00

**Fig 8:**
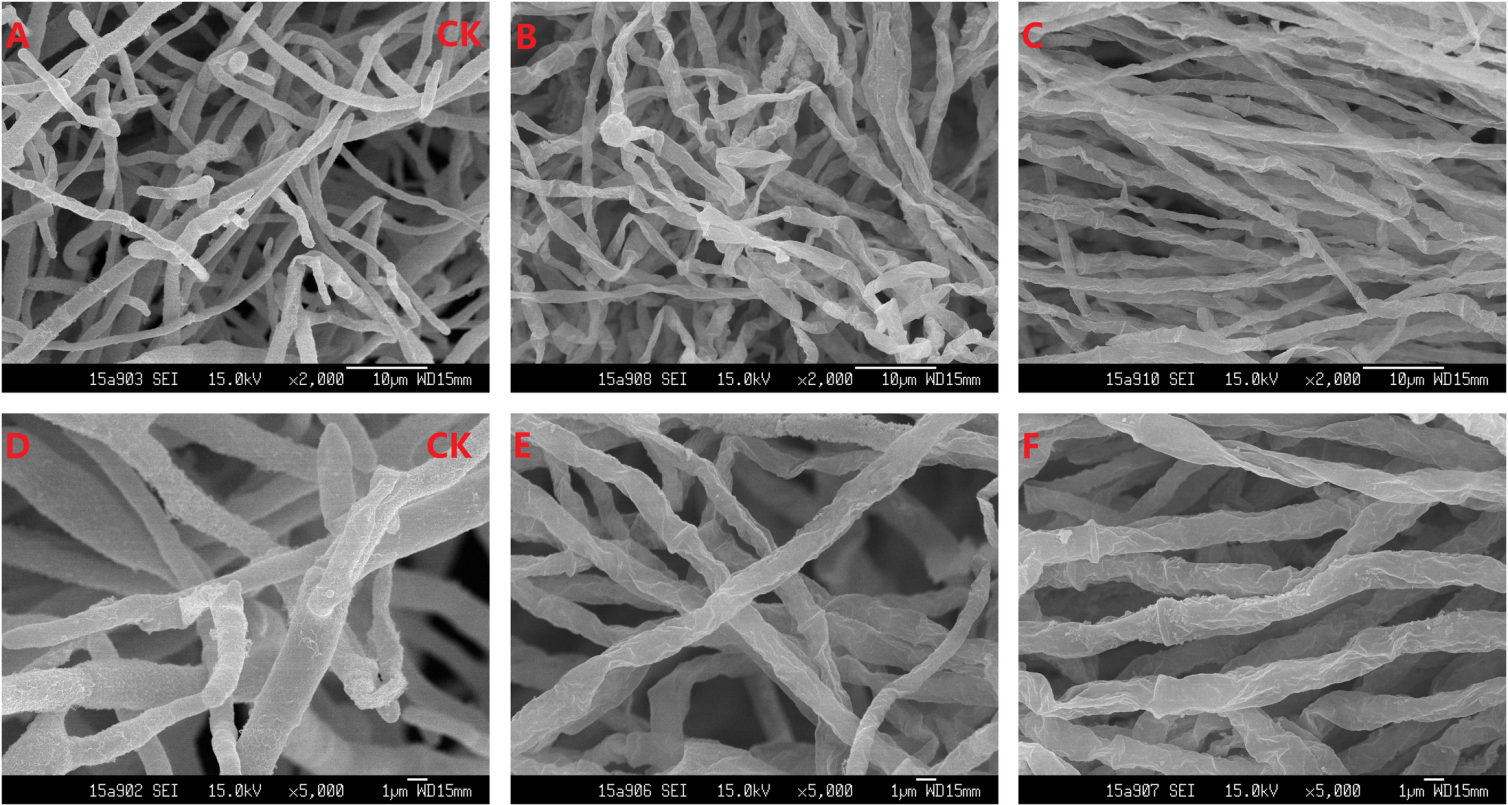
SEM micrographs of *M*. *grisea* mycelia. The concentration of iturin A and bacillomycin F were 62.50 and 125.00 μg/mL, respectively. A and D: mycelia treated without CLPs; B and E: mycelia treated with iturin A; C and F: mycelia treated with bacillomycin F. A, B and C at 2000-fold magnification; D, E and F at 5000-fold magnification.

The surface structures of the hyphae were observed through SEM (Fig 8). The hyphae of the control group (non-treated) grew normally and possessed an intact and smooth surface and a plump tubular structure (Fig 8A and 8B). By contrast, the hyphae of the treated group (62.50 μg/mL iturin A or 125.00 μg/mL bacillomycin F) were damaged. The hyphae treated with iturin A or bacillomycin F were diastrophic and distorted, and their surface were sunk, lumpy, and wrinkled (Fig 8C–8F). The SEM results indicated that bacillomycin F and iturin A could distinctly affect the growth of *M*. *grisea* by disrupting the hyphal surface structure.

## Discussion

Finding alternatives to antibiotics is an important task in modern biotechnology because of the rapid emergence of antibiotic resistance among pathogenic bacteria, which have posed risks not only to the environment but also to consumers[27,28]. Developing new antibiotics has remained in the stagnation phase, and novel antibiotics have been rarely found from natural sources through traditional methods. As such, rapid changes in the strategies used for natural product discovery have been made by developing a cost-effective and high-throughput sequencing technology. This approach has become a fast and inexpensive approach for the identification of “talented” bacteria.

The biological control of pathogenic diseases, such as using antibiotic-producing bacteria, especially *Bacillus* species, to control pathogenic microorganisms, has been extensively explored[29,30]. Compared with conventional chemical antibiotics, antibiotics produced by *Bacillus* provide advantages, including easy degradation without yielding harmful residues[31]. Therefore, *Bacillus*-generated antibiotics are effective and environmentally friendly for the control of pathogenic diseases. *B*. *subtilis* and *B*. *amyloliquefaciens* have been used in commercial biological control products because of their strong antimicrobial activities and high stability under harsh environmental conditions[32,33].

In this research, *B*. *siamensis* JFL15, which exhibited a strong antimicrobial activity against *V*. *harveyi*, *M*. *grisea*, *R*. *solani*, and *C*. *gloeosporioides*, was isolated from the gastrointestinal tract of *T*. *haumela*. On the basis of morphological characteristics, 16S rDNA sequences, and ANI values, we identified this strain as *B*. *siamensis* JFL15. Previous studies revealed that many *Bacillus* species can control pathogens[34]. However, few reports have presented that *Bacillus siamensis* has strong antimicrobial activities against pathogens[35]. Our work is the first to show that *B*. *siamensis* could control *M*. *grisea*, *R*. *solani*, *C*. *gloeosporioides*, and *V*. *harveyi*, which are the main pathogens in rice, mango, and aquaculture, respectively.

The complete genome of *B*. *siamensis* JFL15 was sequenced and then analyzed for the presence of CLP biosynthesis genes. *B*. *siamensis* JFL15 possesses 30 clusters involved in secondary metabolism, but this number is slightly lower than that of *B*. *amyloliquefaciens* FZB42 that comprises 35 gene clusters devoted to secondary metabolism. Of the 30 clusters, 3 were responsible for the biosynthesis of bioactive CLPs via NRPSs: surfactin (*srf*), bacillibactin (*dhb*) and fengycin (*fen*), which corresponded to 78%, 100%, and 100% of the identified gene clusters, respectively. The gene cluster of surfactin biosynthesis was further examined. NRPS product diversity is primarily attributed to the substrates incorporated at the adenylation (A) domain in each module. For example, iturin, mycosubtilin, and bacillomycin belong to the iturin family produced by *Bacillus* NRPSs. However, differences in the A domain of these NRPS clusters leads to incorporation of different building blocks at these domains, resulting in structural variations in the latter parts of iturin, mycosubtilin, and bacillomycin molecules[10]. Seven A domains were present in the cluster of surfactin biosynthesis of *B*. *siamensis* JFL15, and the similarities of A domains in *B*. *siamensis* JFL15 and *B*. *amyloliquefaciens* FZB42 ranged from 95% to 97%. The amino acid backbone structure of the potential peptide was predicted as Glu-Leu-Leu-Val-Asp-Leu-Leu (Fig 5) based on the binding specificities of the A domains. In addition, at least six gene clusters associated with the biosynthesis of bacillaene, difficidin, plantathiazolicin, butirosin, and a putative antibiotic were found in *B*. *siamensis* JFL15 genome.

Six components (a, b, c, d, e, and f) containing 20 compounds with strong antimicrobial activities were isolated and purified from the cell-free supernatants of *B*. *siamensis* JFL15 culture by a combination of Sephadex LH-20 gel filtration chromatography and preparation reversed phase chromatography. Four main types of cyclic lipopeptides are produced by *Bacillus* species: bacillibactin, iturin, fengycin, and surfactin. Two high-purity antifungal components, namely, components b and c, elicited excellent antagonistic effects against *M*. *grisea*, *R*. *solani*, and *C*. *gloeosporioides* compared with botcinins[36]. Components b and c were respectively identified as iturin A and bacillomycin F through LC–MS/MS analysis. ESI-CID-MS/MS analyses indicated that the cyclic peptide was broken to generate a series of specific b- and y-type ion fragments, which could be observed as a “fingerprint” of the MS/MS spectrum of a unique compound. For example, component b was identified as iturin A by the typical (b- or y-type) fragment ions at *m*/*z* 212.1, 392.2, 638.4, 801.4, 915.5, and 932.5, which were consistent with the mass fragments reported in a previous study[25].

Iturin A and bacillomycin F belong to the iturin family, which consists of iturins A, C, D, and E, bacillomycins D, F, L, and LC, bacillopeptin, and mycosubtilin; these substances exhibit strong antifungal activities against various pathogenic fungi[37,38]. In Fig 7C, bacillomycin F was different from iturin A in one of the seven amino acids at position 7, which was replaced by Thr. This study is the first to report that bacillomycin F could control the pathogenic fungi *M*. *grisea* and *R*. *solani* in rice. Although iturins from *B*. *subtilis* and *B*. *amyloliquefaciens* have been extensively investigated[39,40], only a few other *Bacillus* species have been described to have the ability to produce iturins. So far, only two reports have presented that *B*. *siamensis* could secrete CLPs based on standard control[41] or whole-genome analyses[35]. However, CLPs purification and structural identification from the species of *B*. *siamensis* have not been investigated. To the best of our knowledge, this study is the first to purify and identify iturin A and bacillomycin F from the species of *B*. *siamensis*. Moreover, the antifungal activities of these two cyclic lipopeptides against *M*. *grisea*, *R*. *solani*, and *C*. *gloeosporioides* were performed in the present research.

Although compounds of iturin family comprising one iturin A and five bacillomycin Fs were identified from the cell-free supernatants of *B*. *siamensis* JFL15 culture, the complete genome of *B*. *siamensis* JFL15 did not predict a gene cluster involved in iturin biosynthesis. Iturin produced by *B*. *siamensis* JFL15 is possibly bacillomycin F, which is different from iturin A, bacillomycin D, or bacillomycin L produced by other *Bacillus* species.

MIC tests showed that iturin A (component b) and bacillomycin F (component c) exhibited different MICs against various pathogenic fungi (Table 3). Both components had strong antifungal activities, but the antifungal activity of iturin A was slightly higher than that of bacillomycin F. This variation was probably due to the difference in their chemical structures, that is, although the two compounds have the same fatty acid tail (n = 14), bacillomycin F differs from iturin A in one of the seven amino acids at position 7 replaced by Thr, which contains one more methyl (–CH_3_) in its structure compared with that of Ser. Therefore, iturin A might exhibit a high-affinity interaction with the membranes of pathogenic fungi.

The scanning electron microscopy observations demonstrated that the hyphae on the cell surface of *M*. *grisea* underwent severe ultrastructural changes in the presence of iturin A or bacillomycin F, thereby causing a sunk, lumpy, and wrinkled appearance. The cell membrane of *M*. *grisea* hyphae was seriously damaged, suggesting that iturin A and bacillomycin F might exert antifungal activities by changing and penetrating the structure of cell membranes and interacting with intracellular targets, such as other iturin-related antibiotics[24].

In conclusion, a *Bacillus* strain was isolated and identified as *B*. *siamensis* JFL15. Six components containing 20 compounds with strong antagonistic properties against *C*. *gloeosporioides* or *V*. *harveyi* were purified and identified from cell-free supernatants. Our results indicated that *B*. *siamensis* JFL15 may be a promising biocontrol agent for an effective and environmentally friendly control of pathogenic microorganisms. Future studies will apply proteomics and transcriptomics methods to investigate the signaling pathways involved in the antagonistic effects of iturin A and bacillomycin F on their indicator fungi.

## Supporting information

S1 Table Classification, general features and genome sequencing project information for *Bacillus* sp. JFL15 according to the MIGS recommendations. (DOCX)
