# Supplementary material for: Genomics-guided discovery and structure identification of cyclic lipopeptides from the Bacillus siamensis JFL15
Source: PLoS One. 2018 Aug 31;13(8):e0202893. doi: 10.1371/journal.pone.0202893 (PMC6118384; doi:10.1371/journal.pone.0202893)
Supplement: S1 Table — (DOCX) [file pone.0202893.s001.docx]

**S1 Table. Classiﬁcation, general features and genome sequencing project information for *Bacillus* sp. JFL15** **according to the** **MIGS recommendations.**

| **MIGS ID** | **Property** | **Term** |
| --- | --- | --- |
|  | *Current classiﬁcation* | *Domain Bacteria* |
|  |  | Phylum *Firmicutes* |
|  |  | Class *Bacilli* |
|  |  | Order *Bacillales* |
|  |  | Family *Bacillaceae* |
|  |  | Genus *Bacillus* |
|  |  | Specie *Bacillus siamensis* |
|  |  | Strain JFL15 |
|  | Gram stain | Positive |
|  | Cell shape | Rod |
|  | Motility | Motile |
|  | Sporulation | Endospore-forming |
|  | Temperature range | 15-45ºC |
|  | Optimum temperature | 37ºC |
|  | Carbon source | Varied |
|  | Energy source | Heterotrophic |
| **MIGS-6** | Habitat | Gastrointestinal tract of *Trichiurus haumela* |
| **MIGS-6.2** | pH | 5-8 |
| **MIGS-6.3** | Salinity | NaCl 1.0–7.5% |
| **MIGS-22** | Oxygen | Aerobic |
| **MIGS-15** | Biotic relationship | Free living |
| **MIGS-4** | Geographic location | Northern South China Sea |
| **MIGS-5** | Sample collection time | Jan-01,2015 |
| **MIGS-31** | Finishing quality | Improved-high-quality draft |
| **MIGS-28** | Libraries used | Shotgun |
| **MIGS-29** | Sequencing platforms | Illumina HiSeq2000 |
| **MIGS-31.2** | Fold coverage | 160× |
| **MIGS-30** | Assemblers | SOAPdnovo 2.04 |
| **MIGS-32** | Gene calling method | Glimmer 3.0 |
|  | NCBI project ID | 1679193 |
